# Comparative proteomic analysis of glomerular proteins in primary and bucillamine-induced membranous nephropathy

**DOI:** 10.1186/s12014-022-09365-x

**Published:** 2022-07-14

**Authors:** Hajime Kaga, Hirotoshi Matsumura, Takehiro Suzuki, Naoshi Dohmae, Masafumi Odaka, Atsushi Komatsuda, Naoto Takahashi, Hideki Wakui

**Affiliations:** 1 Department of Hematology, Nephrology, and Rheumatology, Akita University Graduate School of Medicine, Akita, Japan; 2 Department of Life Science, Graduate School of Engineering Science, Akita University, 1-1 Tegatagakuen-machi, Akita City, Akita 010-8502 Japan; 3 Biomolecular Characterization Unit, RIKEN Center for Sustainable Research Science, Wako, Japan; 4 Department of Internal Medicine, Ogachi Central Hospital, Yuzawa, Japan

**Keywords:** Bucillamine, Comparative proteomic analysis, Glomerular proteins, Laser microdissection, Mass spectrometry, Membranous nephropathy

## Abstract

**Background:**

Anti-phospholipase A2 receptor autoantibody (PLA2R Ab)-associated membranous nephropathy (MN) is the most common form of primary MN (pMN). On the other hand, bucillamine (BCL), an antirheumatic drug developed in Japan, was reported to cause a rare form of secondary MN (sMN). Between these MN forms, comparative proteomic analysis of glomerular proteins has not been performed.

**Methods:**

We used renal biopsy specimens from 6 patients with PLA2R Ab (+) pMN, 6 patients with PLA2R Ab (‒) pMN, 6 patients with BCL-induced sMN, and 5 control cases (time 0 transplant biopsies). Proteins were extracted from laser-microdissected glomeruli and analyzed using mass spectrometry. The quantification values of protein abundance in each MN group were compared with those in the control group.

**Results:**

More than 800 proteins with high confidence were identified. Principal component analysis revealed a different distribution between the pMN and sMN groups. For further analysis, 441 proteins matched with ≥ 3 peptides were selected. Among the pMN and sMN groups, we compared the profiles of several protein groups based on the structural and functional characteristics, such as immunoglobulins, complements, complement-regulating proteins, podocyte-associated proteins, glomerular basement membrane proteins, and several proteins that are known to be associated with kidney diseases, including MN. In all MN groups, increased levels of immunoglobulins (IgG, IgA, and IgM), complements (C3, C4, and C9), complement factor H-related protein 5, type XVIII collagen, calmodulin, polyubiquitin, and ubiquitin ligase were observed. For some proteins, such as type VII collagen and nestin, the fold-change values were significantly different between the pMN and sMN groups.

**Conclusions:**

Between the pMN and BCL-induced sMN groups, we observed common and different alterations in protein levels such as known disease-associated proteins and potential disease marker proteins.

**Supplementary Information:**

The online version contains supplementary material available at 10.1186/s12014-022-09365-x.

## Background

Membranous nephropathy (MN) is a common cause of nephrotic syndrome (NS) in adults. Histopathological features in MN are subepithelial immune deposits and associated alterations in the glomerular basement membranes (GBM) [1, 2]. After the discovery of circulating antibodies specific for native podocyte antigens, a serology-based diagnostic approach was proposed [1, 2].

Primary MN (pMN), responsible for approximately 70% to 80% of MN cases, is an autoimmune disease mainly caused by circulating anti-phospholipase A2 receptor antibodies (PLA2R Abs) [1, 2]. A predominance of the immunoglobulin (Ig) G4 subclass is characteristic of pMN [1, 2]. Secondary MN (sMN) in the remaining MN cases is caused by infections, drugs, malignancies, or systemic autoimmune diseases [3]. Bucillamine (BCL), a disease-modifying antirheumatic drug developed in 1987 in Japan [4, 5], is widely used for the treatment of rheumatoid arthritis in Japan. This agent has a chemical structure similar to penicillamine and causes sMN [4, 5]. A study of the Japan Renal Biopsy Registry from 2007 to 2015 showed that BCL is the most common causative drug in cases of drug-induced sMN in Japan [6]. A predominance of the IgG4 subclass was not observed in a case-series study from a Japanese institute [4]. This suggests different immunological mechanisms between pMN and BCL-induced sMN.

Renal biopsy is a traditional approach for the definite diagnosis of MN, but it may not be sufficient to establish the true nature of MN [1]. Recent progress in proteomic analysis has furthered our understanding of renal physiological processes [7]. However, in most proteomic approaches to chronic kidney diseases including MN, urine and/or serum samples are analyzed [8]. Recently, Kawata et al. [9] and Ravindran et al. [10] reported the profiles of protein groups, such as Igs, complements, and complement-regulating proteins, in pMN and exostosins-associated sMN using laser-microdissected glomeruli from formalin-fixed paraffin-embedded tissue sections.

In the present study, we performed laser-microdissection and comparative proteomic analysis of glomerular proteins in PLA2R Ab-positive (PLA2R (+)) pMN, PLA2R Ab-negative (PLA2R (−)) pMN, BCL-induced sMN, and control cases. We then characterized the profiles of several protein groups based on the structural and functional characteristics, such as Igs, complements, complement-regulating proteins, podocyte-associated proteins, GBM proteins, and known kidney disease-related proteins, and found different protein profiles between pMN and BCL-induced sMN.

## Methods

### Patients

We enrolled 6 patients with PLA2R (+) pMN, 6 patients with PLA2R (−) pMN, 6 patients with BCL-induced sMN, and 5 healthy transplantation donors (time 0 transplant biopsies) as controls in this study. All patients and healthy donors were Japanese. pMN was diagnosed based on renal biopsy findings and screening for causes of sMN. All patients in the pMN groups did not present with any clinical findings suggesting the causes of sMN [3]. BCL-induced sMN was diagnosed based on renal biopsy findings and clinical course (development of proteinuria after BCL therapy and improvement of proteinuria after discontinuing BCL) [4, 5]. All patients in the BCL-induced sMN group did not present with any clinical findings suggesting the causes of sMN other than BCL exposure [3].

### Clinicopathological analysis

Clinical data of patients and transplantation donors were collected from medical records for age, sex, urinary protein, serum albumin, and creatinine (Cr) at the time of renal biopsy. For patients with BCL-induced sMN, urinary protein levels during BCL therapy and after discontinuing BCL were also collected. NS was defined as urinary protein ≥ 3.5 g/day or g/gCr and hypoalbuminemia (serum albumin ≤ 3.0 g/dL). The estimated glomerular filtration rate (eGFR) was calculated using the formula for Japanese patients [11]. Circulating PLA2R Abs were measured in pMN patients using enzyme-linked immunosorbent assay (ELISA), in-house ELISA, and a standardized commercial ELISA (Euroimmun, Lübeck, Germany), as described previously [12].

Renal biopsy specimens were processed using standard techniques for light, immunofluorescence, and electron microscopy. Formalin-fixed, paraffin-embedded sections were stained with hematoxylin and eosin, periodic acid-Schiff, Masson trichrome, and periodic acid-methenamine silver. Tubulointerstitial lesions (tubular atrophy and interstitial fibrosis) were classified as follows: absent, < 25% (mild), 25–50% (moderate), or > 50% (severe). Cryostat sections for immunofluorescence microscopy were stained with fluorescein isothiocyanate-conjugated rabbit polyclonal antibodies against human IgG, IgA, IgM, κ, λ, C3, and C1q (DakoCytomation, Glostrup, Denmark). Further studies to determine IgG subclasses were performed as described previously [13].

### Laser-microdissection and proteomic analysis

For each case, 8-μm-thick formalin-fixed paraffin-embedded renal biopsy sections were mounted on PEM-Membrane slides (MicroDissect GmbH, Herborn, Germany). Using a Leica LMD7000 laser-microdissection system (Leica Microsystems, Tokyo, Japan), the glomeruli were microdissected and pooled in 0.5-mL microcentrifuge tubes to reach approximately 1,000,000–4,000,000 μm^2^ per case (the total numbers of glomeruli were 58–188). The dissected glomeruli were treated with approximately 100–200 μL of TE Buffer (Promega Corporation, Madison, WI, USA) containing 0.002% hexadecyldimethyl(3-sulfopropyl) ammonium hydroxide inner salt (Tokyo Medical Industry, Tokyo, Kapan). Glomerular proteins were extracted in the microcentrifuge tubes by sonication.

Nano-liquid chromatography-tandem mass spectrometry (nLC-MS/MS) was performed as described previously [14]. In brief, extracted glomerular proteins were subjected to sodium dodecyl sulfate–polyacrylamide gel electrophoresis. The electrophoresis was stopped when the dye front migrated from the stacking gel to the separation gel. The protein bands containing whole proteins were excised and digested with a trypsin in gel. Tryptic digests were quantified by amino acid analysis [15] and 0.01 μg of these were separated on an Easy-nLC 1200 (Thermo Fisher Scientific, Walthman, MA, USA) using a C18 analytical column (NTCC-360/75-3-155, Nikkyo Technos, Tokyo, Japan). The separated peptides were then analyzed on a Q-Exactive HF-X mass spectrometer (Thermo Fisher Scientific) coupled with an Easy-nLC 1200 (Thermo Fisher Scientific).

### Data analysis

Data analysis was performed as described previously [14]. In brief, the data obtained in the nLC-MS/MS experiments were analyzed using Proteome Discover 2.4 (Thermo Fisher Scientific) and Mascot Server 2.7 (Matrix Science; http://www.matrixscience.com/server.html). The National Center for Biotechnology Information non-redundant database (NCBInr) is a predefined database for protein identification. The proteins were quantified by Label free quantification manner according to the Proteome Discoverer User guide (https://assets.thermofisher.com/TFS-Assets/CMD/manuals/Man-XCALI-97808-Proteome-Discoverer-User-ManXCALI97808-EN.pdf). The reproducibility of the data for the samples from each group was confirmed through principal component analysis [16]. The fold-change values obtained during the relative quantification of protein abundance were compared between each MN group and the control group (expressed as PLA2R (+)/C, PLA2R (−)/C, and BCL/C ratios). If the denominator of a fraction was zero or almost zero, the relative protein abundance ratio was defined as 100. If the numerator of a fraction was zero or almost zero, the relative protein abundance ratio was defined as 0.01.

## Results

### Clinicopathological characteristics of MN patients and transplantation donors

The clinicopathological features of our MN patients and transplantation donors are shown in Tables 1 and 2. The median ages of patients with PLA2R (+) pMN, PLA2R (−) pMN, or BCL-induced sMN, and healthy transplantation donors were 63, 78, 45, and 53 years, respectively. There was a female predominance among patients with BCL-induced sMN. In the pMN groups, 10 of the 12 patients developed NS. In the BCL-induced sMN group, 2 of the 6 patients developed NS during BCL therapy and proteinuria improved after BCL discontinuation. Renal function was preserved in all groups.

**Table 1:** Clinical characteristics of patients with membranous nephropathy included in this study and control subjects

	PLA2R (+) MN patients	PLA2R (−) MN patients	BCL-induced MN patients	Transplantation donors
Number of patients and controls	6	6	6	5
Median age (years) (range)	63 (60‒67)	78 (41‒82)	45 (33‒61)	53 (45‒77)
Male:female	3:3	3:3	1:5	1:4
RLA2R antibody titer (positive: ≥ 14 RU/mL)*	36.8 (35.3–576.9)	0 (0–1.5)		
Median proteinuria (g/gCr or g/day) at biopsy (range)	10.0 (5.3‒21.0)	3.7 (2.6 ‒23.7)	0.5 (0.2‒5.5)	0.7 (0.1‒0.9)
Median maximum level during BCL therapy (range)			4.8 (1.7‒8.8)	
Median minimum level after BCL withdrawal (range)			0.2 (0.1‒0.7)	
Median serum albumin (g/dL) (range)	2.2 (1.2‒2.9)	3.0 (1.0‒3.2)	3.1 (2.8‒4.0)	4.3 (3.5‒4.5)
Nephrotic syndrome, *n*	6	4	2	
Median serum Cr (mg/dL) (range)	0.64 (0.49‒0.90)	0.58 (0.39‒0.90)	0.40 (0.40‒0.86)	0.67 (0.60‒0.90)
Median eGFR (mL/min/1.73 m^2^) (range)	72.1 (65.7‒94.4)	99.7 (74.9‒124.6)	131 (66.4‒143.2)	72.1 (66.9‒81.6)

BCL: bucillamine; Cr: creatinine; eGFR: estimated glomerular filtration rate; MN: membranous nephropathy; PLA2R: phospholipase A2 receptor

* Measured by the Euroimmun enzyme-linked immunosorbent assay system

**Table 2:** Pathological characteristics of patients with membranous nephropathy included in this study

	PLA2R (+) MN patients	PLA2R (−) MN patients	BCL-induced MN patients
Median time from presentation to biopsy (months) (range)	2 (1‒4)	2 (1‒24)	2 (1‒3)
Median percentage of global sclerotic glomeruli (range)	13 (4‒23)	4 (0‒6)	6 (0‒20)
TA (absent, mild, moderate, severe), *n*	1, 5, 0, 0	1, 5, 0, 0	2, 4, 0, 0
IFib (absent, mild, moderate, severe), *n*	0, 6, 0, 0	0, 5, 1, 0	1, 3, 2, 0
Glomerular IgG deposition (IF intensities), *n*	4 (2+), 2 (3+)	2 (+), 2 (2+), 2 (3+)	2 (+), 1 (2+), 3 (3+)
IgG1 (IF intensities), *n*	5 (−), 1 (±)	6 (−)	1 (±), 3 (+), 2 (2+)
IgG2 (IF intensities), *n*	3 (−), 2 (+), 1 (2+)	1 (−), 1 (±), 3 (+), 1 (2+)	2 (+), 2 (2+), 2 (3+)
IgG3 (IF intensities), *n*	4 (−), 2 (+)	5 (−), 1 (+)	5 (−), 1 (+)
IgG4 (IF intensities), *n*	2 (+), 1 (2+), 3 (3+)	2 (−), 1 (+), 3 (2+)	3 (−), 1 (±), 2 (+)
Glomerular IgA deposition (IF intensities), *n*	3 (−), 1 (±), 1 (+), 1 (2 +)	1 (−), 2 (±), 2 (+), 1 (2+)	2 (−), 2 (+), 2 (2+)
Glomerular IgM deposition (IF intensities), *n*	5 (−), 1 (+)	2 (−), 3 (±), 1 (+)	1 (−), 2 (±), 2 (+), 1 (2+)
Glomerular κ deposition (IF intensities), *n*	2 (+), 2 (2+), 2 (3+)	1 ( ±), 1 (+), 3 (2+), 1 (3+)	1 ( ±), 2 (+), 1 (2+), 2 (3+)
Glomerular λ deposition (IF intensities), *n*	2 (+), 2 (2+), 2 (3+)	1 ( ±), 1 (+), 3 (2+), 1 (3+)	2 (+), 2 (2+), 2 (3+)
Glomerular C3 deposition (IF intensities), *n*	4 (+), 2 (2+)	1 (−), 1 ( ±), 4 (+)	3 ( ±), 2 (+), 1 (3+)
Glomerular C1q deposition (IF intensities), *n*	6 (−)	5 (−), 1 ( ±)	3 (‒), 2 (+), 1 (2+)
MN stage on electron microscopy (I–IV), *n*	2 (I), 3 (II), 1 (NA)	2 (I), 1 (II), 1 (IV), 2 (NA)	4 (I), 1 (II), 1 (III)

BCL: bucillamine; IF: immunofluorescence; IFib: interstitial fibrosis; Ig: immunoglobulin; MN: membranous nephropathy; NA: not available; PLA2R: phospholipase A2 receptor; TA: tubular atrophy

The median times from presentation to biopsy were 2 months in all groups. The median percentages of global sclerotic glomeruli in the pMN and sMN groups were 4‒13% and 6%, respectively. In most patients in the pMN and sMN groups, tubulointerstitial lesions were absent or mild. Glomerular IgG deposition was observed in all MN patients. IgG4 predominance was observed in the pMN groups, whereas IgG1 deposition was characteristic, in addition to IgG4 deposition, in the BCL-induced sMN group. Co-deposition of IgA, IgM, and C1q was more frequent in the BCL-induced sMN group than in the pMN groups, whereas C3 deposition was noted in most MN patients. MN stages were variable among patients in all groups.

### Comparative proteomic analysis

**Fig. 1:**
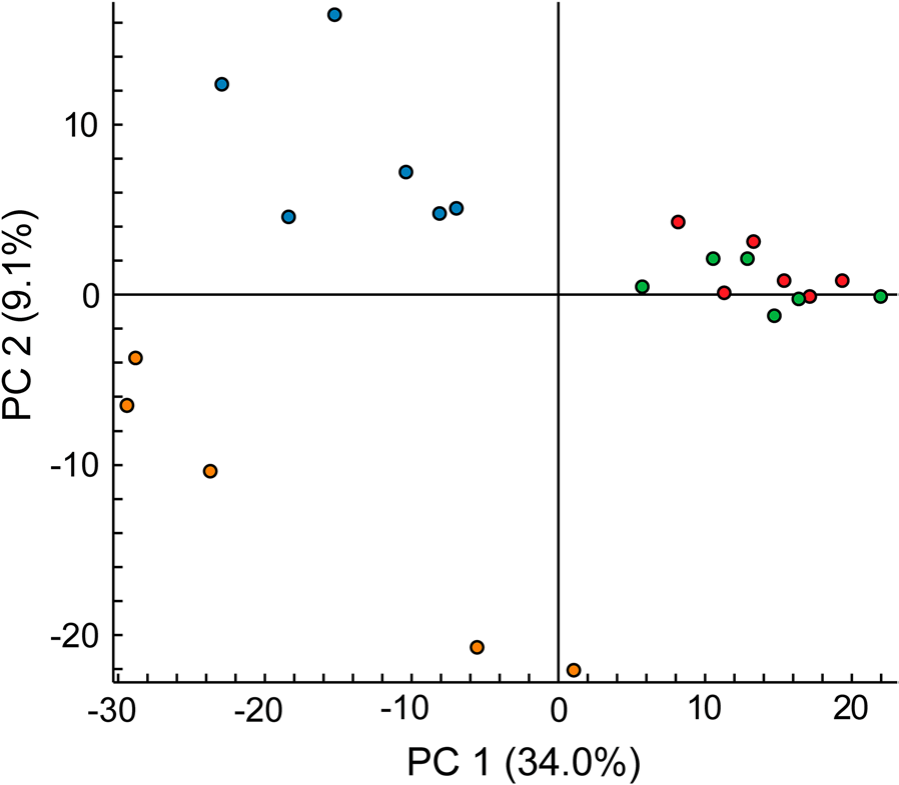
Principle component analysis. The data obtained for 6 patients with PLA2R (+) pMN, 6 patients with PLA2 (−) pMN, 6 patients with BCL-induced sMN, and 5 control patients (time 0 trans plant biopsies) are shown. The data for the PLA2R (+) pMN, PLA2 (−) pMN, BCL-induced sMN, and control groups are represented by red, green, blue, and orange dots, respectively. The proportion of variance captured is given as a percentage for both the first and second principal components (PC1 and PC2)

In our proteomic analysis of glomerular proteins extracted from laser-microdissected glomeruli of the enrolled subjects (Additional file 1: Table S1), principal component analysis revealed a different distribution pattern between the pMN and sMN groups (Fig. 1) (Additional file 2: Table S2). We were able to identify 846 proteins with high confidence (experimental *Q* < 0.01), and further selected 441 proteins that were matched with ≥ 3 peptides. Proteins with increased levels in the MN groups (PLA2R (+)/C, PLAR (‒)/C, and BCL/C ratios of > 2.5) and proteins with decreased levels in the MN groups (PLA2R ( +)/C, PLAR (‒)/C, and BCL/C ratios of < 0.4) are shown in Tables 3, 4, 5, 6 and 7. Proteins that increased or decreased with medium or high confidence (adjusted *P* < 0.05 or < 0.01) are indicated in these tables.

**Table 3:** List of immunoglobulins

Accession ID NCBInr DB	Accession ID GenBank DB	Protein name	Sequence coverage (%)	Peptide match (*n*)	PLA2R (+) pMN patients (*n* = 6) / Transplantation donors (*n* = 5) ratio (Ratio variability [%])	PLA2R (‒) pMN patients (*n* = 6)/ Transplantation donors (*n* = 5) ratio (Ratio variability [%])	BCL-induced sMN patients (*n* = 6)/ Transplantation donors (*n* = 5) ratio (Ratio variability [%])
34527425	AK130586.1	Ig γ1 heavy chain C region	20	7	2.21 (57.87)	2.08 (45.70)	3.25 (87.87)
34535785	AK128421.1	Ig γ2 heavy chain C region	12	5	1.95 (58.20)	3.10 (51.79)	2.44 (57.76)
34535866	AK128477.1	Ig γ4 heavy chain C region	17	6	100 **	100 **	100 **
229537		Ig α heavy chain	12	5	3.48 (79.74)	3.02 (73.42)	2.95 (79.72)
33451	X17115.1	Ig μ heavy chain	9	5	3.04 (77.96)	4.12 (71.01)	3.95 (52.18)
229526		Ig κ light chain	25	3	21.16** (64.07)	12.28* (36.39)	12.33 ** (34.62)
576865216		Ig λ light chain	23	3	1.84 (113.61)	1.84 (104.59)	4.43 (110.45)

BCL: bucillamine; C: constant; DB: database; Ig: immunoglobulin; NCBInr: National Cancer for Biotechnology Information non-redundant; PLA2R: phospholipase A2 receptor; pMN: primary membranous nephropathy; sMN: secondary membranous nephropathy

*P < 0.05, **P < 0.01

**Table 4:** List of complements and complement-regulating proteins

Accession ID NCBInr DB	Accession ID GenBank DB	Protein name	Sequence coverage (%)	Peptide match (*n*)	PLA2R (+) pMN patients (*n* = 6)/Transplantation donors (*n* = 5) ratio (Ratio variability [%])	PLA2R (‒) pMN patients (*n* = 6)/Transplantation donors (*n* = 5) ratio (Ratio variability [%])	BCL-induced sMN patients (*n* = 6)/Transplantation donors (*n* = 5) ratio (Ratio variability [%])
115298678		C3	23	38	7.49 (129.06)	4.98 (135.45)	3.21 (91.36)
443671		C4A	12	17	100**	100**	100**
40737478		C4A3	14	5	100**	100**	100**
119576392		C9	12	7	7.13 (87.45)	7.51 (78.82)	2.69 (85.74)
118442839		CFHR1	14	4		7.36 (68.49)	46.38** (0)
767910533		CFHR5	10	6	100**	100**	100**
578815184		Clusterin	25	13	2.15 (112.32)	2.14 (127.33)	1.49 (98.18)
809019	X05309.1	CR1	3	3	2.05 (54.36)	0.24 (51.06)	4.28 (26.67)
194383558	AK303793.1	C4BPA	13	7	1.79 (106.65)	2.88 (121.81)	1.22 (84.37)
13477169		Vitronectin	18	7	2.72 (92.66)	2.37 (103.65)	2.87 (89.85)

BCL: bucillamine; C4BPA: C4b-binding protein α chain; CFHR: complement factor H-related protein; CR: complement receptor; DB: data base; NCBInr: National Cancer for Biotechnology Information non-redundant; PLA2R: phospholipase A2 receptor; pMN: primary membranous nephropathy; sMN: secondary membranous nephropathy

**P < 0.01

**Table 5:** List of podocyte-associated proteins

Accession ID NCBInr DB	Accession ID GenBank DB	Protein name	Sequence coverage (%)	Peptide match (*n*)	PLA2R (+) pMN patients (*n* = 6)/Transplantation donors (*n* = 5) ratio (Ratio variability [%])	PLA2R (−) pMN patients (*n* = 6)/Transplantation donors (*n* = 5) ratio (Ratio variability [%])	BCL-induced sMN patients (*n* = 6)/Transplantation donors (*n* = 5) ratio (Ratio variability [%])
150170672		THSD7A	2	3	0.97 (83.71)	1.80 (80.92)	2.05 (92.14)
204306657		Nephrin	3	4	0.73 (60.28)	0.81 (61.73)	0.98 (38.71)
7657615		Podocin	9	3	1.59 (75.95)	1.82 (73.38)	2.55 (94.26)
1034591669		Zo-1	8	12	1.51 (78.11)	1.81 (68.22)	1.20 (78.79)
5453599		F-actin-capping protein α2	18	4	4.55 (62.71)	4.92 (65.91)	1.67 (72.65)
578810794		Synaptopodin	15	11	1.36 (69.45)	1.30 (61.22)	1.54 (66.93)
12025678		α-actinin-4	58	44	1.68 (100.17)	1.62 (95.46)	1.09 (91.42)
12667788		Myosin-9	11	19	1.87 (85.85)	1.87 (87.83)	1.15 (78.52)
219520307	BC143318.1	Podocalyxin	9	5	2.33 (86.65)	3.21 (79.78)	1.86 (97.71)
119615053		Integrin α3	6	6	0.85 (66.75)	1.01 (72.04)	1.35 (81.96)
76879682		Nestin	30	28	8.40 (95.45)	0.46 (136.13)	0.01**
62414289		Vimentin	80	59	3.10 (107.54)	2.48 (106.48)	1.23 (107.57)

BCL: bucillamine; DB: data base; NCBInr: National Cancer for Biotechnology Information non-redundant; PLA2R: phospholipase A2 receptor; pMN: primary membranous nephropathy; sMN: secondary membranous nephropathy; THSD7A: thrombospondin type-1 domain-containing 7A; Zo-1: zonula occludens-1

**P < 0.01

**Table 6:** List of glomerular basement membrane proteins

Accession ID NCBInr DB	Accession ID GenBank DB	Protein name	Sequence coverage (%)	Peptide match (*n*)	PLA2R (+) pMN patients (*n* = 6)/Transplantation donors (*n* = 5) ratio (Ratio variability [%])	PLA2R (−) pMN patients (*n* = 6)/Transplantation donors (*n* = 5) ratio (Ratio variability [%])	BCL-induced sMN patients (*n* = 6)/Transplantation donors (*n* = 5) ratio (Ratio variability [%])
125987809		COL4A1	6	5	0.15* (120.23)	0.07** (91.69)	0.38 (100.14)
116256354		COL4A2	5	6	0.77 (47.23)	0.71 (65.75)	0.56 (57.53)
119591260		COL4A3	7	6	0.87 (102.70)	1.00 (87.83)	1.39 (74.61)
116256356		COL4A4	4	4	0.56 (105.95)	1.12 (83.70)	1.08 (68.49)
1034673478		COL4A5	4	4	1.98 (62.44)	1.58 (81.23)	1.48 (72.75)
20147503		Laminin α5	19	61	0.80 (95.43)	0.82 (92.06)	0.83 (98.88)
1103585		Laminin β2	30	46	1.01 (74.85)	1.06 (80.03)	0.94 (85.94)
145309326		Laminin γ1	17	25	0.73 (80.56)	0.92 (77.70)	0.81 (89.79)
119590445		Nidogen-1	21	27	1.24 (67.88)	1.23 (67.35)	1.20 (62.93)
530360311		Agrin	28	43	0.80 (99.26)	1.00 (100.63)	0.97 (91.60)
11602963		HSPG perlecan	15	49	1.12 (82.16)	1.07 (90.05)	1.06 (87.98)

BCL: bucillamine; COL: collagen; DB: data base; HSPG: heparan sulfate proteoglycan; NCBInr: National Cancer for Biotechnology Information non-redundant; PLA2R: phospholipase A2 receptor; pMN: primary membranous nephropathy; sMN: secondary membranous nephropathy

*P < 0.05, **P < 0.01

**Table 7:** List of glomerular proteins other than proteins listed in Tables 3, 4, 5, 6

Accession ID NCBInr DB	Accession ID GenBank DB	Protein name	Sequence coverage (%)	Peptide match (*n*)	PLA2R (+) pMN patients (*n* = 6)/Transplantation donors (*n* = 5) ratio (Ratio variability [%])	PLA2R (−) pMN patients (*n* = 6)/Transplantation donors (*n* = 5) ratio (Ratio variability [%])	BCL-induced sMN patients (*n* = 6)/Transplantation donors (*n* = 5) ratio (Ratio variability [%])
119585300		COL7A1	1	3	0.54 (83.65)	1.33 (129.12)	15.51** (92.1)
215274264		COL18A1	6	9	3.27 (100.40)	3.15 (93.18)	2.74 (108.70)
61680528		Calmodulin	42	6	100**	100**	100:**
2627129		Polyubiquitin	32	4	4.70 (102.34)	4.60 (87.45)	2.65 (90.06)
47825361		FBOX50 (ubiquitin ligase)	12	3	3.36 (39.01)	3.76 (46.07)	3.23 (75.70)

BCL: bucillamine; COL: collagen; DB: data base; FBOX50: F-box only protein 50 (now known as ubiquitin ligase); NCBInr: National Cancer for Biotechnology Information non-redundant; PLA2R: phospholipase A2 receptor; pMN: primary membranous nephropathy; sMN: secondary membranous nephropathy

**P < 0.01

Igs are summarized in Table 3. Ig γ1, Ig γ2, Ig γ4, Ig α, and Ig μ heavy chains, and Ig κ and Ig λ light chains were detected. Increased levels of Ig γ4, Ig α, and Ig μ heavy chains, and Ig κ light chain were found in all MN groups. Levels of Ig γ1 and Ig γ2 heavy chains, and Ig λ light chain also increased (> 1.8) in all MN groups.

Complements and complement-regulating proteins are summarized in Table 4. Complement C3, C4A, C4A3, and C9 were detected. These complement proteins increased in all MN groups. Complement factor H-related proteins (CFHR1 and CFHR5), clusterin, complement receptor 1, C4b-binding protein α chain, and vitronectin were also detected. Among them, CFHR5 was abundant in all MN groups, whereas CFHR1 was abundant in the BCL-induced sMN group.

Detected podocyte-associated proteins [1, 2, 17, 18], including thrombospondin type-1 domain-containing 7A, nephrin, podocin, zonula occludens-1, F-actin capping protein α2 (CapZ) [19], synaptopodin, α-actinin-4, myosin-9, podocalyxin [20], integrin α3, nestin [21, 22], and vimentin [21], are summarized in Table 5. In the PLA2R Ab (+) pMN group, CapZ, nestin, and vimentin were abundant. Lower levels of nestin were observed in the BCL-induced sMN group.

Detected GBM proteins [23], including type IV collagen α chains, laminin chains, nidogens-1, agrin, and heparan sulfate proteoglycan 2 (HSPG 2), are summarized in Table 6. Lower levels of type IV collagen α1 chain were observed in all MN groups. The levels of the other proteins were almost unchanged.

Other glomerular proteins, including type VII collagen α1 chain [24], type XVIII collagen α1 chain [25], calmodulin [26], polyubiquitin [27], and F-box only protein 50 (previously known as non-specific cytotoxic cell receptor protein 1, now known as FBXO50 ubiquitin ligase) [28], are summarized in Table 7. Among them, the levels of type XVIII collagen α1 chain, calmodulin, polyubiquitin, and FBXO50 ubiquitin ligase increased in all MN groups. The levels of type VII collagen α1 chain increased in the BCL-induced sMN group.

## Discussion

Kawata et al. [9] and Ravindran et al. [10] recently reported the proteomic profiles of Igs, complement proteins, and complement-regulating proteins in pMN and exostosins-associated sMN using laser-microdissected glomeruli and mass spectrometry. In the present study, we performed comparative proteomic analysis of glomerular proteins extracted from laser-microdissected glomeruli of PLA2R (+) pMN, PLA2R (−) pMN, and BCL-induced sMN patients and transplantation donors.

In a study by Kawata et al. [9], post-transplant kidneys (at the 1-h biopsy samples after reperfusion of the blood) were used as the controls. They also reported that glomerular structural proteins and podocyte-related proteins were detected as the major proteins in pre-transplant kidneys (at the 0-h biopsy samples after perfusion with preservation solution) [9]. In a study by Ravindran et al. [10] and in the present study, time 0 biopsies were used as the controls. As discussed below, similar results regarding Igs, complement proteins, and complement-regulating proteins, were obtained in these studies.

In studies by Kawata et al. [9] and Ravindran et al. [10], peptide identifications were accepted at greater than 95% probability, and the proteins identified had at least 2 matching peptides. In our proteomic analysis, principal component analysis demonstrated a different distribution between the pMN and sMN groups (Fig. 1). More than 800 proteins with high confidence (experimental *Q* < 0.01) were identified, and 441 proteins matched with ≥ 3 peptides were further selected for more precise analysis. In addition to Igs, complement proteins, and complement-regulating proteins, we listed podocyte-associated proteins, GBM proteins, and other glomerular proteins that are known to be associated with kidney diseases including MN. After analyzing the abundance of these proteins, proteins in each MN group were compared with those in the control group.

Regarding Igs (Table 3), Ig γ1, Ig γ2, Ig γ4, Ig α, and Ig μ heavy chains, and Ig κ and Ig λ light chains were detected. Increased levels of these Ig heavy and light chains were found in all MN groups. This suggests that polyclonal co-deposits of IgG, IgA, and IgM are detected by nLC-MC/MS in all MN groups. In a study by Ravindran et al. [10], increased levels of Ig γ1, Ig γ2, and Ig γ4 heavy chains were also observed in their patients with PLA2R (+) pMN and exostosins-associated sMN. Although pMN is considered an IgG4-dominant disease, PLA2R Abs of other IgG subclasses are present in the majority of patients with PLA2R-associated MN [29].

Regarding complement proteins and complement-regulating proteins (Table 4), the observed levels were largely consistent with those in pMN reported by Kawata et al. [9] and in PLA2R-associated pMN and exostosins-associated sMN reported by Ravindran et al. [10] except for CFHR1. Increased levels of C3, C4A, C9, and CFHR5 were also observed in all MN groups. This suggests common activation mechanisms in the complement cascade and regulatory pathways between pMN and BCL-induced sMN.

In our proteomic analysis, main podocyte-associated proteins were detected (Table 5). Among them, increased levels of the actin capping protein CapZ [19] were observed in the pMN groups. As CapZ contributes to the formation and maintenance of a specialized cell–cell contacts between podocyte foot processes and barrier function [19], increased CapZ may have a protective role in injured podocytes. Regarding podocyte nestin expression, there were controversial findings in the puromycin aminonucleoside-induced rat nephrosis model and diseased human kidneys [21, 22]. Upregulation of podocyte nestin was observed in the rat model [21], whereas podocyte nestin expression was reduced in patients with proteinuria [22]. In our study, the level of nestin increased in PLA2R (+) pMN, whereas it decreased in BCL-induced sMN. The reason for this difference is not clear at present.

Main BGM proteins [23] were also detected in our proteomic analysis (Table 6). In all MN groups, the levels of type IV collagen, laminins, nidogen-1, agrin, HSPG2 were unaltered, except for type IV collagen α1 chain. As mature GBM are composed of type IV collagen α3α4α5 chains [23], decreased type IV collagen α1 chain may have a minor role in GBM assembly in MN. Glomerular abnormalities can be classified into 4 stages in MN [2]. As the MN stages in our patients were variable in all MN groups, BGM protein components may be preserved even at advanced stages.

We further listed possible disease-associated proteins in MN (Table 7). Among them, an increased level of type VII collagen was found in BCL-induced sMN. Increased expression of type VII collagen was also observed in several glomerular diseases in the previous study [24]. On the other hand, increased levels of type XVIII collagen α1 chain were found in all MN groups. In the previous study of mice lacking type XVIII collagen α1 chain, podocyte foot process effacement was observed [25]. Up-regulation of this type of collagen chain in MN may play a role in structural maintenance in injured podocytes. Regarding signaling proteins, increased levels of calmodulin were observed in all MN groups. This may be associated with the direct effects of Ca^2+^/calmodulin on actin filament formation [26] in injured podocytes. Regarding the ubiquitin–proteasome system [30], increased levels of polyubiquitin [27] and FBXO50 ubiquitin ligase [28] were noted in all MN groups, suggesting upregulation of the ubiquitin–proteasome system in podocytes in response to injury in MN.

Despite the power of proteomics profiles and use of bioinformatics analyses, there are several limitations to our work. We only performed nLC-MS/MS on a few glomeruli samples, and there are significant differences in age and clinical severity of disease between the pMN and sMN groups. Patients with sMN were younger than patients with pMN. Patients with sMN showed mostly sub-nephrotic proteinuria and higher eGFR. Some proteomic findings in this study, such as differences in type IV collagen α1 chain reduction in the pMN group compared to the sMN group, may be reflected by different clinical characteristics [31]. It is necessary to increase the accuracy and reduce the variability of our analyses through further verification in a large sample size.

## Conclusions

This is the first report of comparative proteomic analysis of glomerular proteins extracted from laser-microdissected glomeruli in pMN and BCL-induced sMN. Principal component analysis demonstrated a different distribution between the pMN and sMN groups. Between these MN groups, we observed common and different alterations in protein levels such as known disease-associated proteins and potential disease marker proteins. Our results suggest common and different pathogenetic mechanisms between pMN and BCL-induced sMN.

## Supplementary Information

**Additional file 1: Table S1.** Summary of proteomics data.

**Additional file 2: Table S2.** List of proteins in the top and bottom 10 of the loading score of PC1 and PC2.

## Data Availability

Please contact the corresponding author for data requests.

## Abbreviations

BCL: Bucillamine
CapZ: Capping actin protein of muscle Z-line
CFHR: Complement factor H-related protein
Cr: Creatinine
eGFR: Estimated glomerular filtration rate
ELISA: Enzyme-linked immunosorbent assay
FBXO: F-box only protein
GBM: Glomerular basement membrane
HSPG: Heparan sulfate proteoglycan
Ig: Immunoglobulin
MN: Membranous nephropathy
NCBInr: National Center for Biotechnology Information non-redundant database
nLC-MS/MS: Nano-liquid chromatography-tandem mass spectrometry
NS: Nephrotic syndrome
PLA2R Abs: Anti-phospholipase A2 receptor antibodies
pMN: Primary membranous nephropathy
sMN: Secondary membranous nephropathy
