# Supplementary material for: Comparative proteomic analysis of glomerular proteins in primary and bucillamine-induced membranous nephropathy
Source: Clin Proteomics. 2022 Jul 14;19:26. doi: 10.1186/s12014-022-09365-x (PMC9281048; doi:10.1186/s12014-022-09365-x)
Supplement: Supplementary file 2 — Additional file 2: Table S2. List of proteins in the top and bottom 10 of the loading score of PC1 and PC2. [file 12014_2022_9365_MOESM2_ESM.docx]

**Supplementary Table S2. List of proteins in the top and bottom 10 of the loading score of PC1 and PC2**

**Top 10 proteins of loading score of PC1**

| Accession | Protein Description | PC 1 (34.0%) | PC 2 (9.1%) |
| --- | --- | --- | --- |
| 15778930 | ARP2 actin-related protein 2 homolog (yeast) [Homo sapiens] | 0.0574 | 0.0087 |
| 1040205135 | Chain A, Crystal Structure Of The Fusion Protein Linked By Rhoa And The Gap Domain Of Mgcracgap | 0.0566 | 0.0252 |
| 197692221 | 14-3-3 protein beta/alpha [Homo sapiens] | 0.0566 | -0.0063 |
| 119608187 | hCG1780554 [Homo sapiens] | 0.0564 | 0.0120 |
| 308818195 | dihydropyrimidinase-related protein 2 isoform 1 [Homo sapiens] | 0.0564 | -0.0097 |
| 119571516 | ribosomal protein L23a, isoform CRA_a [Homo sapiens] | 0.0560 | 0.0041 |
| 530389143 | PREDICTED: plectin isoform X3 [Homo sapiens] | 0.0560 | -0.0003 |
| 62088634 | Heterogeneous nuclear ribonucleoproteins C1/C2 variant [Homo sapiens] | 0.0560 | -0.0140 |
| 635576376 | Chain A, 14-3-3-gamma In Complex With Influenza Ns1 C-terminal Tail Phosphorylated At S228 | 0.0558 | -0.0094 |
| 189053128 | unnamed protein product [Homo sapiens] | 0.0554 | -0.0250 |

**Bottom 10 proteins of loading score of PC1**

| Accession | Protein Description | PC 1 (34.0%) | PC 2 (9.1%) |
| --- | --- | --- | --- |
| 31873640 | hypothetical protein [Homo sapiens] | -0.0485 | 0.0012 |
| 15988456 | Chain A, Refined Solution Structure Of Human Cystatin | -0.0483 | 0.0232 |
| 119581085 | keratin 10 (epidermolytic hyperkeratosis; keratosis palmaris et plantaris), isoform CRA_b [Homo sapiens] | -0.0481 | -0.0157 |
| 13562132 | catalase [Homo sapiens] | -0.0477 | -0.0033 |
| 7592966 | histamine N-methyltransferase [Homo sapiens] | -0.0473 | 0.0200 |
| 13435361 | desmocollin-1 isoform Dsc1a preproprotein [Homo sapiens] | -0.0463 | -0.0229 |
| 341838586 | MHC class I antigen, partial [Homo sapiens] | -0.0461 | -0.0130 |
| 767999450 | PREDICTED: serpin B12 isoform X1 [Homo sapiens] | -0.0444 | -0.0188 |
| 16117380 | gastric cancer-related protein FKSG9 [Homo sapiens] | -0.0440 | 0.0151 |
| 1034699597 | PREDICTED: LOW QUALITY PROTEIN: killer cell immunoglobulin-like receptor 2DS3 isoform X1 [Homo sapiens] | -0.0439 | -0.0284 |

**Top 10 proteins of loading score of PC2**

| Accession | Protein Description | PC 1 (34.0%) | PC 2 (9.1%) |
| --- | --- | --- | --- |
| 7662010 | zinc finger protein 516 [Homo sapiens] | 0.0015 | 0.0896 |
| 112694863 | immunoglobulin heavy chain variable region [Homo sapiens] | 0.0084 | 0.0874 |
| 47825361 | F-box only protein 50 [Homo sapiens] | 0.0025 | 0.0834 |
| 215274264 | RecName: Full=Collagen alpha-1(XVIII) chain; Contains: RecName: Full=Endostatin; Flags: Precursor | 0.0087 | 0.0827 |
| 119576392 | complement component 9, isoform CRA_b [Homo sapiens] | 0.0124 | 0.0776 |
| 700652946 | immunoglobulin gamma heavy chain variable region, partial [Homo sapiens] | 0.0139 | 0.0765 |
| 222418587 | NACHT and WD repeat domain-containing protein 2 [Homo sapiens] | -0.0016 | 0.0765 |
| 767913370 | PREDICTED: arf-GAP with SH3 domain, ANK repeat and PH domain-containing protein 2 isoform X1 [Homo sapiens] | -0.0061 | 0.0753 |
| 229149 | hemoglobin beta | 0.0025 | 0.0736 |
| 10637464 | immunoglobulin kappa chain variable region [Homo sapiens] | 0.0152 | 0.0727 |

**Bottom 10 proteins of loading score of PC2**

| Accession | Protein Description | PC 1 (34.0%) | PC 2 (9.1%) |
| --- | --- | --- | --- |
| 56789516 | GRHPR protein, partial [Homo sapiens] | -0.0182 | -0.1004 |
| 157881389 | Chain A, Structure Of The Aflatoxin Aldehyde Reductase In Complex With Nadph | -0.0191 | -0.1001 |
| 109148552 | keratin, type II cytoskeletal 3 [Homo sapiens] | -0.0260 | -0.0949 |
| 1335064 | fibrillin [Homo sapiens] | -0.0199 | -0.0943 |
| 189069169 | unnamed protein product [Homo sapiens] | -0.0149 | -0.0941 |
| 23110925 | proteasome subunit beta type-6 isoform 1 precursor [Homo sapiens] | -0.0171 | -0.0922 |
| 16579888 | fructose-1,6-bisphosphatase 1 [Homo sapiens] | -0.0100 | -0.0902 |
| 13129092 | transmembrane protein 109 precursor [Homo sapiens] | 0.0149 | -0.0898 |
| 530381843 | PREDICTED: glutathione S-transferase A1 isoform X1 [Homo sapiens] | -0.0035 | -0.0885 |
| 595582373 | beclin-2 [Homo sapiens] | -0.0283 | -0.0883 |
